# Increased Adherence to the Mediterranean Diet after Lifestyle Intervention Improves Oxidative and Inflammatory Status in Patients with Non-Alcoholic Fatty Liver Disease

**DOI:** 10.3390/antiox11081440

**Published:** 2022-07-25

**Authors:** Margalida Monserrat-Mesquida, Magdalena Quetglas-Llabrés, Cristina Bouzas, Sofía Montemayor, Catalina M. Mascaró, Miguel Casares, Isabel Llompart, Lucía Ugarriza, J. Alfredo Martínez, Josep A. Tur, Antoni Sureda

**Affiliations:** 1 Research Group in Community Nutrition and Oxidative Stress, University of the Balearic Islands-IUNICS, 07122 Palma de Mallorca, Spain; margalida.monserrat@uib.es (M.M.-M.); m.quetglas@uib.es (M.Q.-L.); cristina.bouzas@uib.es (C.B.); sofiamf16@gmail.com (S.M.); c.mascaro@uib.es (C.M.M.); isabel.llompart@ssib.es (I.L.); luciaugarriza@gmail.com (L.U.); antoni.sureda@uib.es (A.S.); 2 Health Research Institute of Balearic Islands (IdISBa), 07120 Palma de Mallorca, Spain; 3 CIBER Fisiopatología de la Obesidad y Nutrición (CIBEROBN), Instituto de Salud Carlos III (ISCIII), 28029 Madrid, Spain; 4 Radiodiagnosis Service, Red Asistencial Juaneda, 07011 Palma de Mallorca, Spain; casaresmiguel@gmail.com; 5 Clinical Analysis Service, University Hospital Son Espases, 07198 Palma de Mallorca, Spain; 6 Camp Redó Primary Health Care Center, 07010 Palma de Mallorca, Spain; 7 Cardiometabolics Precision Nutrition Program, IMDEA Food, CEI UAM-CSIC, 28049 Madrid, Spain; jalfredo.martinez@imdea.org; 8 Department of Nutrition, Food Sciences, and Physiology, University of Navarra, 31008 Pamplona, Spain

**Keywords:** Mediterranean diet, fatty liver, aerobic capacity, oxidative stress, inflammation

## Abstract

Background: A Mediterranean diet (MedDiet) is recommended as a therapy for non-alcoholic fatty liver disease (NAFLD) because there is no specific pharmacological treatment for this disease. Objective: To assess the relationship between the adherence to the Mediterranean diet and the intrahepatic fat content (IFC), levels of oxidative stress, and inflammation biomarkers after a 6-month lifestyle intervention in NAFLD patients. Methods: Patients diagnosed with NAFLD (n = 60 adults; 40–60 years old) living in the Balearic Islands, Spain, were classified into two groups, according to the adherence to the MedDiet after 6 months of lifestyle intervention. Anthropometry, blood pressure, IFC, maximal oxygen uptake, and pro/antioxidant and inflammatory biomarkers were measured in plasma and in PBMCs before and after the intervention. Results: Reductions in weight, body mass index, IFC, blood pressure levels, circulating glucose, glycosylated hemoglobin, and markers of liver damage—aspartate aminotransferase (AST), alanine aminotransferase (ALT), gamma-glutamyl transferase (GGT), and cytokeratin 18 (CK-18)—were observed after the intervention. The highest reductions were observed in the group with the best adherence to the MedDiet. A significant improvement in cardiorespiratory fitness was also observed in the group with a higher adherence. The activities of catalase in plasma and catalase and superoxide dismutase in blood mononuclear cells increased only in the group with a higher adherence, as well as the catalase gene expression in the blood mononuclear cells. The plasma levels of malondialdehyde and myeloperoxidase decreased, and resolvin-D1 increased in both groups after the intervention, whereas interleukin-6 levels decreased only in the group with a higher adherence to the MedDiet. Conclusions: A greater adherence to the MedDiet is related to greater improvements in IFC, cardiorespiratory fitness, and pro-oxidative and proinflammatory status in NAFLD patients after a 6-month nutritional intervention based on the MedDiet.

## 1. Introduction

The Mediterranean diet (MedDiet) is characterized by a high intake of vegetables, legumes, cereals, olive oil, nuts, fish, and dairy products; low quantities of meat; and an optional moderate wine consumption [1,2]. Previous studies established the MedDiet as an adequate treatment to reduce the incidence of major cardiovascular events and for the management of type 2 diabetes mellitus (T2DM) [3,4]. In this sense, a recent meta-analysis concluded that adherence to the MedDiet is inversely related to the risk of overweight and/or obesity, which is of practical importance for public health [5]. Moreover, it has been described that the MedDiet can reduce liver fat and it is recommended as a primary treatment for the management of non-alcoholic fatty liver disease (NAFLD) because there is no specific pharmacological treatment against this disease [6,7]. In this sense, the pharmacological treatment of risk factors that are commonly associated with the metabolic syndrome may also contribute to the improvement of NAFLD [8]. Although there is some evidence supporting the beneficial effects of some pharmacological agents, to date, there is no formally approved therapy for this disorder. NAFLD is characterized by the excessive fat accumulation in the liver parenchyma not related to alcohol abuse [9]. The development of NAFLD, if not properly treated and managed, can progress to steatohepatitis, fibrosis, or even cirrhosis and hepatocarcinoma [10,11,12]. Furthermore, the consequences of NAFLD are related to other metabolic diseases, such as hypertension, high levels of triglycerides, lower levels of high-density lipoprotein (HDL)-cholesterol, overweight or obesity, and insulin resistance [13]. Lately, suffering NAFLD and metabolic syndrome (MetS) at the same time is defined as a new concept known as metabolic dysfunction-associated fatty liver disease (MAFLD) [14].

Oxidative stress, defined as an imbalance between the cellular levels of the antioxidant and the pro-oxidants, is related to many diseases, especially with an inflammatory process [15]. The increase in pro-oxidant agents, mainly reactive oxidative species (ROS) and reactive nitrogen species (RNS), could cause oxidative damage to biomolecules and alter the normal functioning of cells [16]. Regarding the liver, oxidative stress was implicated in the pathogenesis of NAFLD and NASH, and also directly associated with the progression and severity of the disease [17,18]. Among the mechanisms that contribute to the prevention of oxidative stress and maintenance of cellular homeostasis, the antioxidant system is essential to avoid oxidative damage to cell components, such as lipids, proteins, and DNA [19]. High intrahepatocyte fat causes lipotoxicity that alters mitochondrial metabolic pathways and the redox status, inducing endoplasmic reticulum stress, damaging the liver structure and hepatic function [20]. In NAFLD, the lipotoxicity and oxidative stress induced by the excess in both triglycerides and free fatty acids also contribute to inflammation [19,21]. In this inflammatory process, Kupffer cells, macrophages resident in the hepatic sinusoids, play a central role in exhibiting a change into a proinflammatory phenotype (M1), releasing proinflammatory cytokines [22].

Considering that nutritional intervention has been related to an improvement in the anthropometric and metabolic parameters, the aim of this study was to assess the relationship between the adherence to the MedDiet and the intrahepatic fat contents (IFC) as a primary endpoint, and the levels of oxidative stress and inflammation biomarkers as secondary endpoint, after a 6-month lifestyle intervention in NAFLD patients.

## 2. Materials and Methods

### 2.1. Design and Participants

A total of 67 adults from Mallorca, Balearic Islands, Spain, were included in this study. These participants were a subset of the “Prevention and Reversion of NAFLD in Obese Patients with Metabolic Syndrome by Mediterranean Diet and Physical Activity (FLIPAN)” clinical trial (ClinicalTrials.gov Identifier: NCT04442620; https://clinicaltrials.gov/ct2/show/NCT04442620; accessed on 22 February 2022). They were selected considering the following inclusion criteria: aged between 40 and 60 years old, diagnosis of NAFLD by magnetic resonance imaging (MRI), body mass index (BMI) between 27 and 40 kg/m^2^, and at least three of the five criteria of metabolic syndrome (MetS) according to the International Diabetes Federation (IDF) consensus [23], which are:

(1) Waist circumference of ≥ 102 cm in men and ≥ 88 cm in women.
(2) Serum glucose level ≥ 100 cm.
(3) Systolic blood pressure ≥ 130 mmHg or diastolic blood pressure ≥85 mmHg.
(4) Triglycerides levels ≥ 150 mg/dL.
(5) Reduced HDL-cholesterol levels < 40 mg/dL in men and < 50 mg/dL in women.

The study protocol followed the ethical standards of the Declaration of Helsinki, and all procedures were approved by the Ethics Committee of the Balearic Islands (ref. IB 2251/14 PI). All participants were informed of the purpose and implications of the study, and all gave their written consent to participate. As specified in the consent form, participation was voluntary, and participants had the right to withdraw from the study at any time.

### 2.2. FLIPAN Clinical Trial Guidelines

The adults who met the selection criteria were randomized following dietary interventions previously described for 6 months, characterized by an energy reduction of 25–30% of baseline calories intake and increase energy expenditure by 400 kcal/70 kg (5.7 kcal per kg of body weight) [24]. Trained dietitians provided patients a daily calorie prescription, food regimens based on exchange systems, and a seven-day meal for each season by trained dietitians.

Information about intakes was collected at baseline and 6 months, using a validated 148-items Food Frequency Questionnaire (FFQ) [25]. The 148 items consist of usual portion sizes of foods and drinks with response categories to indicate frequency of consumption over a period of 12 months. Participants were asked how often, on average, they consumed the amount of item reported on the FFQ during the past year and responded using nine options ranging from never or less than once per month to six or more times per day. Additional foods not included in the questionnaire and the frequency of consumption were manually entered. Energy and nutrients intakes were calculated by multiplying the nutrient composition of the portion size of each item by the frequency of consumption using a computer program based on available food composition tables [26]. Dietary information derived from the 148-items FFQ included total energy expressed as kcal per day (kcal/d), macro- and micro-nutrient intakes, and intakes according to food groups.

Adherence to the Mediterranean diet (AMD) was assessed at baseline and 6 months by means of a validated 17-items questionnaire [27]. A score was given for each met objective: 1 (compliance) or 0 (non-compliance). The total score ranged between 0 and 17, such as a score of 0 indicated no compliance and a score of 17 indicated maximum adherence.

The median value was calculated by subtracting the median value of AMD at 6 months from baseline, and subjects were distributed in “under median value (<50%)” (n = 31) and “above median value (≥50%)” (n = 36). Participants were classified into two groups according to the variation in the score obtained by a questionnaire to assess the adherence to AMD at the beginning and at the end of the intervention (baseline and 6-month follow-up).

### 2.3. Anthropometrics and Clinical Assessment

Weight (kg) was measured using a Segmental Body Composition Analyser for impedance testing (Tanita MC780P-MA, Tanita, Tokyo, Japan) with barefoot subjects and light clothes, subtracting 0.6 kg. BMI was calculated by dividing the weight in kilograms by the square of height in meters. Height was determined with an anthropometer (Seca 214, SECA Deutschland, Hamburg, Germany) to the closest millimeter. Blood pressure was measured with a semi-automatic oscillometer (Omron HEM, 705CP, Hoofddorp, The Netherlands) in the arm, registering the highest diastolic blood pressure, taking three measures, and waiting for 1 min between each determination. Intrahepatic fat contents (IFC) were performed with a 1.5-T MRI (Signa Explorer 1.5T, General Electric Healthcare, Chicago, IL, USA) by using a 12-channel phased-array coil [28]. The maximal oxygen uptake (VO_2_max) was determined with Chester step test [29]. Data on antidiabetic and antihypertensive drug intake was also collected.

### 2.4. Biochemical Parameters and Hemogram

Venous blood samples were collected, after 12-h overnight fasting conditions, from the antecubital vein in suitable vacutainers with ethylenediaminetetraacetic acid (EDTA) as an anticoagulant. Biochemical parameters: glucose, glycosylated hemoglobin (Hb1ac), triglycerides (TG), high-density lipoprotein-cholesterol (HDL-cholesterol), low-density lipoprotein-cholesterol (LDL-cholesterol), total cholesterol, aspartate aminotransferase (AST), alanine aminotransferase (ALT), gamma-glutamyl transferase (GGT), and c-reactive protein (CRP) were measured using standardized clinical procedures. The hematological parameters (hematocrit) and cell counts (erythrocytes, leukocytes, platelets, neutrophils, lymphocytes, monocytes, eosinophils, and basophils) were determined in whole blood by an automatic flow cytometer analyzer (Technion H2, VCS system, Bayer, Leverkusen, Germany).

### 2.5. Plasma and PBMCs Isolation

Plasma samples were obtained by centrifuging whole fresh blood at 1700× *g* for 15 min at 4 °C. The Peripheral Blood Mononuclear Cells (PBMCs) fraction was purified from fresh whole blood and isolated following the described protocol of Separation of White Blood Cells [30], using the reagent Ficoll-Paque PLUS (GE Healthcare Bio-Sciences AB, Uppsala, Sweden) [31].

### 2.6. Enzymatic Determinations

The antioxidant enzymes catalase (CAT) and superoxide dismutase (SOD) were measured in plasma and PBMCs, using a Shimadzu UV-2100 spectrophotometer (Shimadzu Corporation, Kyoto, Japan) at 37 °C. CAT activity was determined by the spectrophotometric method of Aebi based on the decomposition of H_2_O_2_ [32], whereas SOD activity was measured by an adaptation of the method of Flohe and Otting based on the inhibition of the reduction of cytochrome C by superoxide anion [33].

### 2.7. Measurement of tGSH and GSSG

Total glutathione (tGSH) and glutathione oxidized (GSSG) were measured in blood samples, using a Shimadzu UV-2100 spectrophotometer (Shimadzu Corporation, Kyoto, Japan) at 37 °C. Cells were disrupted with 6% of metaphosphoric acid and incubated for 20 min at 4 °C. Then, the samples were centrifugated at 15,600× *g* for 10 min. After that, the supernatants were put in graduated glass tubes and neutralized with 15% Na_3_PO_4_ to pH 7.5. For determination of tGSH, aliquots of the neutralized samples were estimated spectrophotometrically at 412 nm and following DNTB reduction in the presence of 50 mM NADPH and glutathione reductase. GSSG determination was estimated adding 400 μL of samples to 8 μL of vinil piridine and left at room temperature for 40 min. After that, samples were estimated spectrophotometrically, using the procedure described above. Two calibration curves of standard GSH and GSSG were set up under the same conditions. The tGSH/GSSG ratio was calculated by dividing the concentrations of tGSH and GSSG.

### 2.8. RNA Extraction and Real-Time PCR

mRNA expression of Toll-Like Receptor 4 (TLR4), catalase (CAT), glutathione peroxidase (GPX), copper zinc superoxide dismutase (Cu,ZnSOD), manganese superoxide dismutase (MnSOD), nuclear factor erythroid 2-related factor 2 (Nrf2) was determined by Real-Time PCR based on the incorporation of a fluorescent reporter dye and using human 18S ribosomal as the reference gene. For this purpose, total RNA was isolated from PBMCs by extraction with Tripure^®^ (Tripure Isolation Reagent, Roche Diagnostics, Mannheim, Germany). Then, according to the manufacturer’s instructions, 1 µg of RNA from each sample was reverse transcribed using TaqMan Reverse Transcription Reagents (Life Technologies^®^, Vall Allen Way Carlsbad, CA, USA) for 60 min at 42 °C and 5 min at 99 °C in a 10 µL final volume. The 3 µL resulting cDNA was amplified using the Light-Cycler^®^ 480 SYBR^®^ Green I Master (Roche Diagnostics, Mannheim, Germany). Target cDNAs were amplified as follows: 10 min, 95 °C followed by 45 cycles of amplification, using LightCycler^®^ 96. The primer sequence and amplification conditions are presented in Table 1. Baseline under median value patients was taken as a reference group and referred to as 1.

### 2.9. Immunoassays

Myeloperoxidase (MPO) (Cusabio^®^ Technology Llc, Houston, TX, USA), Resolvin D1 (Cayman Chemical^®^, Ann Arbor, MI, USA), Irisin (Cell Biolabs^®^, San Jose, CA, USA), and Cytokeratin 18 (CK-18) (PEVIVA^®^, Diapharma Group, Inc., West Chester, OH, USA) were measured using ELISA kits, following the manufacturer’s instructions. Interleukin-6 (IL-6) and Tumor necrosis factor-alpha (TNFα) levels were estimated using Human Custom ProcartaPlexTM (Invitrogen by Thermo Fisher Scientific, Bender MedSystems GmbH, Vienna, Austria), following the guidelines for use. All immunoassays were carried out in plasma samples.

### 2.10. Statistics

The Statistical Package for Social Sciences (SPSS v.28 for Windows, IBM Software Group, Chicago, IL, USA) was used for statistical analysis. Data were obtained at baseline and after 6 months Results are represented as the mean ± standard error of the mean (SEM), and the level of significance was established at *p* < 0.05 for all statistics. The statistical significance of the data was checked by two-way analysis of variance (ANOVA) after adjustment for adherence to Mediterranean diet (AMD) and time (T). Furthermore, the analysis of each variable was adjusted by baseline values of the analyzed variable. A Bonferroni post hoc test was carried out when significant differences were found between groups.

## 3. Results

The median value obtained for the adherence to the MedDiet at the beginning of the intervention for all subjects was 8, while in the end, it was increased to 12. The subjects improved 4 points in adherence to the MedDiet, which was used to classify them into two groups, above and below this value. Table 2 shows a summary of the participants’ hypertension and diabetes treatment.

### 3.1. Anthropometric and Biochemical Parameters

Table 3 shows evidence of the significant differences in the ALT levels between the baseline groups. Moreover, significant differences were observed in weight, BMI, systolic and diastolic blood pressure, glucose, HbA1c, AST, ALT, and GGT when comparing the evolution after 6 months. The participants after the 6-month follow-up in the above median value group showed lower values in BMI, systolic blood pressure, AST, and ALT than the baseline. The GGT levels in both groups after 6 months showed differences with the baseline. The glucose levels were lower in the group above the median value than those in the under median group after 6 months. No differences were evidenced in triglycerides, HDL-cholesterol, LDL-cholesterol, total cholesterol, and CRP.

**Figure 1:**
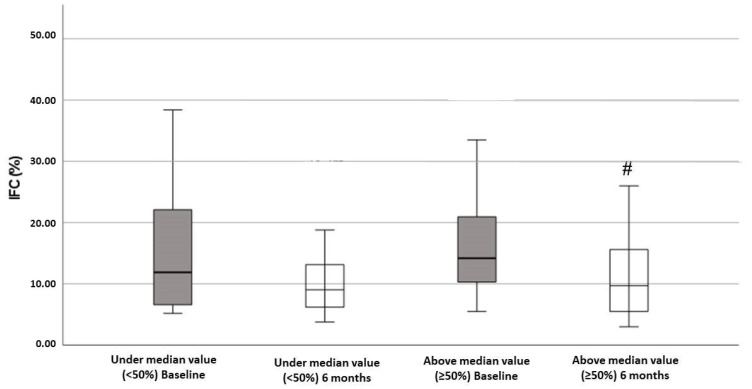
Intrahepatic fat contents (IFC) of patients in this study, classified according to adherence to Mediterranean diet (AMD). Significant improvement of IFC was found after 6 months of follow-up in above median value group (≥50%). Statistics: Two-way ANOVA. Results are presented as mean ± SEM. # Difference in means between time.

Figure 1 showed the levels of IFC according to the degree of improvement in adherence to the MedDiet. The IFC levels decreased after the intervention period, but this decrease was only significant in the group with better improvement in the adherence to the MedDiet.

**Figure 2:**
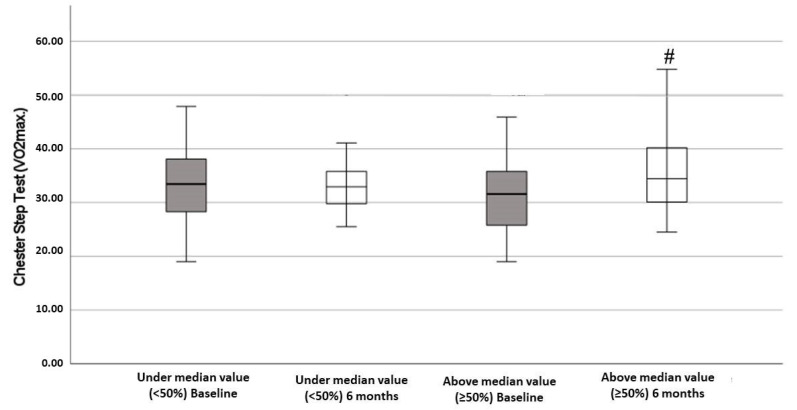
Chester step test (VO_2_max.) of patients in this study, classified according to adherence to Mediterranean diet. Significant improvement of Chester step test was found after 6-month follow-up in above median value group (≥50%). Statistics: Two-way ANOVA. Results are presented as mean ± SEM. # Difference in means between time.

Figure 2 showed the results of VO_2_max determined with the Chester step test, according to the degree of improvement in the adherence to the MedDiet. The obtained data reported a significant improvement after 6 months of the intervention with respect to the baseline in the group above the median value.

### 3.2. Hematological Parameters

Table 4 reports significant differences in the hematocrit, neutrophils, basophils, and eosinophils levels, whereas no differences were described in the other hematological variables. At the end of the intervention period, the hematocrit was higher in the group above the median than the group below, whereas the neutrophil and basophil counts were lower. The number of circulating eosinophils decreased after the intervention period only in the group with a higher adherence to the MedDiet.

### 3.3. Oxidative Stress Biomarkers

Table 5 shows the changes in enzymatic activities in plasma and PBMCs, as well as in mRNA expression in PBMCs. The activity of plasma CAT remained unchanged after the 6 months of intervention, whereas the activity of SOD in plasma significantly increased in the group with a higher adherence. The enzymatic activities of CAT and SOD in PBMCs increased after 6-months intervention, but the differences were only significant in the group above the median value. TLR4, CAT, MnSOD, and Nrf2 relative mRNA expressions were assessed in PBMCs. The mRNA levels of TLR4 showed a significant decrease after the 6-month intervention in the group above the median value. The mRNA levels of CAT significantly increased after 6 months in the group above the median value compared to the baseline. No differences were found in MnSOD and Nrf2 gene expression.

Table 6 represents the tGSH and GSSG levels and the tGSH/GSSG ratio in blood. A significant increase in tGSH and the tGSH/GSSG ratio were demonstrated in the above median value group after 6 months intervention.

### 3.4. Immuno-Assays

Table 7 shows the changes in the plasma levels of MPO, resolvin D1, irisin, CK-18, IL-6, and TNFα. The levels of MPO, CK-18, and IL-6 levels were significantly decreased after 6 months of intervention in the group above the median, whereas the levels of Resolvin D1 increased. No changes were observed in the Irisin and TNFα plasma levels.

## 4. Discussion

### 4.1. Strengths and Limitations

There is limited scientific evidence on the effect of adherence to the Mediterranean diet and the intrahepatic fat contents (IFC), and levels of oxidative stress and inflammation biomarkers after a 6-month lifestyle intervention in NAFLD patients; then, the first strength of the present study is that it contributes to increasing knowledge about it. The longitudinal design provides more evidence than cross-sectional designs. The standardized protocol followed reduces the risk of information bias. On top of the strengths, our findings could be easily implemented into clinical practice. This strength is very important because there is no existing alternative treatment for NAFLD yet [6,7]. Nonetheless, the present study has some limitations. Firstly, the main limitation would be the small sample size. A bigger sample size could give a more confident answer to which intervention was the best. Secondly, people in the FLIPAN trial were between 40–60 years old, and this is a limitation when it comes to extrapolating results to all of the population. Thirdly, there is a selection bias in baseline patients; this fact is because, at the beginning of this study, the groups are classified according to their diet group. Then, no signs were shown with this classification, and for this reason, it has been decided to classify according to the adherence to the MedDiet, and with this selection, homogeneity is lost a bit. Lastly, although the diagnosis was made by MRI, having been able to do biopsies could have more accurately assessed the evolution of steatosis.

### 4.2. Anthropometric and Biochemical Parameters

The main findings of this study are that higher increases in the adherence to the MedDiet after the 6-month nutritional intervention are clearly related to a better oxidative stress and inflammatory state in patients with NAFLD, which may be useful to treat this disease. Patients with a higher adherence also showed better evolution in the pathology with lower IFC and circulating levels of CK-18. Previous studies showed that a higher adherence to the MedDiet was associated with reduced NAFLD prevalence and insulin resistance and with lower severe liver disease among patients with NAFLD [34,35].

Present results are in accordance with previous studies that evidenced a bodyweight loss and reduced changes in systolic and diastolic blood pressure after increasing the adherence to the MedDiet [36,37]. Like these results, it was reported that a low adherence to the MedDiet was associated with high blood glucose and HbA1c levels in diabetic patients [38,39]. These results confirm the fact that the MedDiet contributes to an improvement in glycemic control and insulin sensitivity and a reduction in the incidence of cardiovascular events. AST, ALT, and GGT are widely used as markers of liver injury and may be useful measures for monitoring NAFLD [40]. The better evolution in these enzymes in the group with a higher adherence to the MedDiet agrees with previous findings that evidenced a significant improvement between the baseline and end-treatment in patients with NAFLD after MedDiet follow-up, reducing the risk and severity of NAFLD, as well as the levels of circulating liver enzymes [7,41]. Moreover, the plasma levels of CK-18, an intracellular protein associated with necrosis and apoptosis of hepatocytes [42], decreased mainly in the group with better improvement in the MedDiet. A previous study observed an association between high levels of CK-18 and hypertension in NAFLD patients; therefore, CK-18 could be helpful to detect early hypertension [43]. These results highlight the beneficial effects of the MedDiet by observing a decrease in plasma liver damage markers in the group that has most improved their adherence to the MedDiet.

Adherence to the MedDiet is associated with better antioxidant capacity and anti-inflammatory and antifibrotic status due to the increased consumption of monounsaturated fatty acids, omega-3, and antioxidant compounds [44,45]. In fact, a meta-analysis has shown that omega-3 fatty acids were negatively associated with hepatic steatosis [46]. Moreover, it was demonstrated that an antioxidant-enriched MedDiet contributes to the restoration of normal lipid metabolism, which could prevent NAFLD development and progression [47]. In the present study, the beneficial effects of the nutritional intervention were evidenced by a significant reduction in IFC after 6 months of consuming the MedDiet. Similarly, an inverse association between the ADM and liver damage was observed in NAFLD patients due to a reduction in intrahepatic lipids detected with magnetic resonance [48]. These patients who most improved their diet quality also significantly increased their physical capacity as assessed by the Chester step test. These results suggest the existence of an association between diet quality and physical exercise practice, contributing more efficiently to improve the NAFLD, agreeing with previously published results [49]. These results support the conclusions of a previous meta-analysis that a high adherence to the MedDiet was associated with better physical performance and global cognitive function [50].

### 4.3. Hematological Parameters

Regarding hematological parameters, lower levels of neutrophils, eosinophils, and basophils were observed in the group above the median after a 6-month follow-up, while platelets, although they tend to decrease, did not present statistical differences. These immune cell types are granulocytes responsible for innate immunity and are also considered markers of inflammation. The decrease in their number in the group with a higher adherence may be related to better biochemical parameters and lower fat accumulation that would reduce the proinflammatory state of patients and the mobilization of immune cells. It is in accordance with previous evidence of a significant reduction in leukocyte number after 12 weeks of nutritional intervention with the MedDiet and extra virgin olive oil (EVOO) [51]. A significant reduction was also observed in leukocytes, neutrophils, and lymphocytes after sustained weight loss at 2 years [52]. Moreover, in healthy subjects, an inverse relation between the adherence to the MedDiet and the food antioxidant content with white blood cell counts and platelets was observed [53].

### 4.4. Oxidative Stress and Inflammatory Biomarkers

In the present study, the patients with the best increase in their adherence to the MedDiet presented with enhanced antioxidant in plasma and PBMCs and better redox status after the intervention period. The beneficial effects of the MedDiet are attributed, among other factors, to the presence of bioactive compounds with antioxidant and anti-inflammatory activities [54,55]. In fact, a nutritional intervention based on the MedDiet pattern increased the total antioxidant capacity (TAC) of serum compared to the control group assigned to a low-fat diet [56]. In addition to this direct action, some components of the MedDiet can induce endogenous antioxidant in metabolic syndrome patients [57,58]. Although the expression of CuZnSOD, MnSOD, and Nrf2 also tended to increase its variation, it was not significant. This lack of differences could derive from the variability between individuals; thus, increasing the number of participants could have shown clearer differences. However, the expression of the GPX as downstream genes induced by Nrf2 increased significantly in the higher adherence group [59]. In addition, the high levels of GSH after 6 months of intervention could also derive from the activation of Nrf2 because genes responsible for the synthesis of GSH are also induced by Nrf2.

MPO is released by neutrophils and monocytes in inflammatory conditions, enhancing this inflammatory process and inducing oxidative stress. High levels of MPO were observed in obese subjects, mainly if they simultaneously presented higher values of hs-CRP with respect to normal-weight subjects [60]. Moreover, high MPO activity was also reported in liver biopsies from patients with fatty liver simple steatosis, progressively increasing from NAFLD to NASH [61]. In the present study, the greater reduction observed in the group with a better adherence after a 6-month nutritional intervention may be due to a better inflammatory profile that decreases both the number of circulating immune cells and their pre-activation state.

Resolvin D1 is a pro-revolving mediator of inflammation derived from docosahexaenoic acid, which can contribute to the improvement of obesity-related metabolic dysfunctions, reducing endoplasmic reticulum stress [62]. Previously, it was demonstrated that resolvin D1 levels were lower in patients with a higher IFC than in subjects with lower fat accumulation [63]. The results of this study revealed that participants from both groups, after a 6-month follow-up, showed higher levels of resolvin D1 than the baseline values, indicating that even small improvements in the adherence to the MedDiet have beneficial anti-inflammatory effects. In accordance with the present results, it was evidenced that stimulated neutrophils from patients with metabolic syndrome increased the release of resolvins after the weight loss program [64].

On the contrary, the levels of TNFα and IL-6 as proinflammatory markers show a tendency to decrease, although it is only significant for IL-6 in the group with the highest adherence. Previous data reported an association between these inflammatory biomarkers with impairment of oxidative status due to NAFLD and fat accumulation [65,66]. This decrease in inflammatory cytokines agrees with the decrease in TLR4 expression. Activation of TLRs induces a cascade of intracellular signaling pathways leading to proinflammatory cytokine production and can also contribute to insulin resistance [67]. TLR4 can be activated by saturated fatty acids and ceramides which could be relevant in NAFLD [68]. In fact, the expression of TLR4 is higher in the liver of NAFLD patients than in healthy patients [69]. Weight loss, together with a better metabolic profile, can reduce the proinflammatory state and the presence of free fatty acids, reducing the expression of TLR4 and its downstream products.

Irisin is a cytokine mainly induced by exercise involved in the regulation of glucose metabolism and energy homeostasis. An increment of irisin levels in circulation was associated with a more aggressive phenotype of liver disease [70]. However, and although a tendency to decrease its levels has been observed after 6 months of follow-up with respect to the initial values, the differences were not significant.

## 5. Conclusions

NAFLD is a common chronic liver disorder that is related to excessive accumulation of fats in the liver with increasing prevalence and no effective pharmacological treatment. The present study showed that an improvement in the dietary quality, evaluated by the adherence to the MedDiet, can improve the anthropometric and biochemical parameters, as well as the IFC and the physical capacity of the patients, which agrees with previous published results [49]. A better adherence to the MedDiet also improved the oxidative and proinflammatory status in patients with NAFLD after a 6-month lifestyle intervention. Although the subjects followed similar recommendations, the results obtained highlighted that a greater adherence to the marked guidelines allows them to have a better evolution of NAFLD.

## Figures and Tables

**Table 1:** Primer sequence and conditions used in Real-Time PCRs.

Gene	Gene Accession No.	Primer	Conditions	
18S	NM_001002.4	Fw: 5′-ATgTgAAgTCACTgTgCCAg Rv: 5′-gTgTAATCCgTCTCCACAgA	95 °C 60 °C 72 °C	10 s 10 s 15 s
TLR4	NM_138554.5	Fw: 5′-ggTCACCTTTTCTTgATTCCA Rv: 5′-TCAgAggTCCATCAAACATCAC	95 °C 60 °C 72 °C	10 s 10 s 15 s
CAT	NM_001752.4	Fw: 5′-TTTggCTACTTTgAggTCAC Rv: 5′-TCCCCATTTgCATTAACCAg	95 °C 60 °C 72 °C	10 s 10 s 15 s
GPX	NM_000581.4	Fw: 5′-TTC CCg TgC AAC Cag TTT g Rv: 5′-TTC ACC TCg CAC TTC TCg AA	95 °C 63 °C 72 °C	10 s 10 s 15 s
CuZnSOD	NM_000454.5	Fw: 5′-TCAggAgACCATTgCATCATT Rv: 5′-CgCTTTCCTgTCTTTgTACTTTCTTC	95 °C 63 °C 72 °C	10 s 10 s 15 s
MnSOD	NM_000636.4	Fw: 5′-CgTgCTCCCACACATCAATC Rv: 5′-TgAACgTCACCgAggAgAAg	95 °C 60 °C 72 °C	10 s 10 s 12 s
Nrf2	NM_006164.5	Fw: 5′-gCgACggAAAgAgTATgAgC Rv: 5′-gTTggCAgATCCACTggTTT	95 °C 60 °C 72 °C	10 s 10 s 15 s

Fw: Forward; Rv: Reverse; TLR4: Toll-Like Receptor 4; CAT: catalase; GPX: glutathione peroxidase; Cu,ZnSOD: copper zinc superoxide dismutase; MnSOD: manganese superoxide dismutase; Nrf2: nuclear factor erythroid 2-related factor 2.

**Table 2:** Summaries of the characteristics of the population “Baseline” and “6 months” stratified by adherence to Mediterranean diet.

	Under Median Value (<50%)		Above Median Value (≥50%)	
	Baseline (n = 31)	6 months (n = 31)	Baseline (n = 36)	6 months (n = 36)
Not receive hypertension treatment	60%	58.6%	58.3%	51.4%
Receive hypertension treatment	40%	41.1%	41.7%	48.6%
Not receive diabetes treatment	60%	58.6%	83.3%	82.9%
Receive diabetes treatment	40%	41.4%	16.7%	17.1%

**Table 3:** Anthropometric and biochemical characteristics of participants “Baseline” and “6 months” stratified by adherence to Mediterranean diet.

	Under Median Value (<50%)		Above Median Value (≥50%)		ANOVA		
	Baseline (n = 31)	6 Months (n = 31)	Baseline (n = 36)	6 Months (n = 36)	AMD	T	AMD × T
	Mean ± SEM	Mean ± SEM	Mean ± SEM	Mean ± SEM			
Weight (kg)	95.3 ± 2.0	91.9 ± 1.0	94.2 ± 1.7	88.7 ± 1.5	0.220	0.011	0.541
BMI (kg/m^2^)	34.2 ± 0.5	33.1 ± 0.5	33.3 ± 0.5	31.4 ±0.4 #	0.004	0.002	0.429
Systolic blood pressure (mmHg)	134.3 ± 2.3	133.8 ± 2.0	140.4 ± 1.8	132.9 ± 1.5 #	0.166	0.035	0.070
Diastolic blood pressure (mmHg)	80.3 ± 0.9	80.5 ± 0.9	83.3 ± 1.1	81.6 ± 0.9	0.038	0.440	0.297
Glucose (mg/dL)	116.6 ± 4.9	117.9 ± 6.0	107.4 ± 2.0	101.8 ± 2.0 *	0.001	0.581	0.381
HbA1c (%)	6.15 ± 0.15	5.94 ± 0.11	5.87 ± 0.07	5.71 ± 0.06	0.009	0.066	0.857
Triglycerides (mg/dL)	250.1 ± 49.1	184.5 ± 13.5	187.9 ± 7.7	168.8 ± 13.5	0.119	0.090	0.352
HDL-cholesterol (mg/dL)	42.7 ± 1.5	43.8 ± 1.6	41.2 ± 0.7	43.5 ± 1.1	0.473	0.182	0.637
LDL-cholesterol (mg/dL)	124.7 ± 3.9	126.4 ± 4.1	127.4 ± 4.0	121.7 ± 4.2	0.809	0.628	0.358
Total Cholesterol (mg/dL)	214.0 ± 7.9	204.9 ± 4.8	206.1 ± 4.6	196.6 ± 4.7	0.144	0.096	0.974
AST (U/L)	23.8 ± 0.9	22.0 ± 0.7	26.0 ± 0.8	23.6 ± 0.9 #	0.022	0.015	0.703
ALT (U/L)	31.4 ± 2.1	25.4 ± 1.6	38.5 ± 2.2 *	30.7 ± 1.6 #	0.001	<0.001	0.630
GGT (U/L)	44.1 ± 3.4	35.5 ± 2.7#	43.7 ± 2.6	34.8 ± 2.4 #	0.843	0.002	0.943
CRP (mg/dL)	0.51 ± 0.06	0.42 ± 0.04	0.51 ± 0.07	0.38 ± 0.06	0.735	0.065	0.818

Abbreviations: BMI, body mass index; Hb1Ac, glycated hemoglobin 1A; HDL-cholesterol, high density lipoprotein; LDL-cholesterol, low density lipoprotein; AST, aspartate aminotransferase; ALT, alanine aminotransferase; GGT, gamma glutamyl transferase; CRP, c-reactive protein; SEM: standard error media. Results are expressed as mean ± SEM. Statistics by two-way ANOVA: AMD effect of adherence to the Mediterranean diet, T effect of time, AMD × T interaction between adherence to the Mediterranean diet and time. * Difference in means between participants under median value and above median value (AMD). # Difference in means between time. Each variable was adjusted by baseline values of the analyzed variable.

**Table 4:** Hemogram of participants “Baseline” and “6 months” stratified by adherence to Mediterranean diet (AMD).

	Under Median Value (<50%)		Above Median Value (≥50%)		ANOVA		
	Baseline (n = 31)	6 Months (n = 31)	Baseline (n = 36)	6 Months (n = 36)	AMD	T	AMD × T
	Mean ± SEM	Mean ± SEM	Mean ± SEM	Mean ± SEM			
Haematocrit (%)	43.4 ± 0.5	43.1 ± 0.4	44.3 ± 0.4	44.8 ± 0.3 *	<0.001	0.777	0.289
Erythrocytes (10^6^/μL)	4.91 ± 0.06	4.92 ± 0.05	4.96 ± 0.04	5.00 ± 0.04	0.131	0.515	0.686
Leukocytes (10^3^/μL)	7.44 ± 0.18	7.31 ± 0.20	7.24 ± 0.23	6.81 ± 0.21	0.087	0.180	0.478
Platelets (10^3^/μL)	236.7 ± 5.9	236.5 ± 6.3	230.8 ± 5.1	222.8 ± 5.8	0.088	0.477	0.501
Neutrophils (10^3^/μL)	4.09 ± 0.12	4.02 ± 0.13	3.82 ± 0.15	3.57 ± 0.15 *	0.010	0.245	0.529
Lymphocytes (10^3^/μL)	2.43 ± 0.07	2.28 ± 0.07	2.55 ± 0.10	2.44 ± 0.08	0.090	0.111	0.789
Monocytes (10^3^/μL)	0.61 ± 0.02	0.59 ± 0.02	0.63 ± 0.03	0.56 ± 0.02	0.999	0.066	0.352
Eosinophils (10^3^/μL)	0.26 ± 0.02	0.22 ± 0.01	0.25 ± 0.02	0.19 ± 0.01 *	0.002	0.199	0.481
Basophils (10^3^/μL)	0.058 ± 0.003	0.052 ± 0.003	0.062 ± 0.003	0.054 ± 0.003 #	0.253	0.027	0.946

Results are expressed as mean ± SEM. Statistics by two-way ANOVA: AMD effect of adherence to the Mediterranean diet, T effect of time, AMD × T interaction between adherence to the Mediterranean diet and time. * Difference in means between participants under median value and above median value (AMD). # Difference in means between time. Each variable was adjusted by baseline values of the analyzed variable.

**Table 5:** Oxidative stress biomarkers in PBMCs and plasma of participants “Baseline” and “6-month intervention” stratified by adherence to Mediterranean diet (AMD).

	Under Median Value (<50%)		Above Median Value (≥50%)		ANOVA		
	Baseline (n = 31)	6 Months (n = 31)	Baseline (n = 36)	6 Months (n = 36)	AMD	T	AMD × T
	Mean ± SEM	Mean ± SEM	Mean ± SEM	Mean ± SEM			
Plasma activity							
CAT (kat/L blood)	50.6 ± 2.1	47.9 ± 3.8	49.3 ± 1.6	45.2 ± 3.0	0.458	0.206	0.775
SOD (pkat/L blood)	288.2 ± 9.0	307.9 ± 9.9	282.0 ± 8.9	305.4 ± 5.4 #	0.606	0.011	0.823
PBMCs activity							
CAT (kat/10^9^ cells)	149.0 ± 18.7	153.7 ± 8.6	120.9 ± 8.4	173.2 ± 8.3 #	0.702	0.012	0.036
SOD (nkat/10^9^ cells)	148.3 ± 13.7	163.2 ± 10.6	102.3 ± 10.7	180.9 ± 9.7 #	0.251	<0.001	0.010
PBMCs mRNA expression							
TLR4 (%)	100.0 ± 12.9	92.8 ± 10.9	97.7 ± 12.7	64.0 ± 8.0 #	0.157	0.042	0.227
Nrf2 (%)	100.0 ± 16.3	101.8 ± 15.5	85.5 ± 12.3	112.2 ± 16.5	0.906	0.398	0.461
CAT (%)	100.0 ± 19.2	183.7 ± 18.9 #	87.8 ± 10.7	179.4 ± 24.8 #	0.724	<0.001	0.865
GPX (%)	100.0 ± 15.8	97.6 ± 9.0	100.8 ± 10.9	168.9 ± 23.4 #*	0.042	0.064	0.047
Cu,ZnSOD (%)	100.0 ± 23.4	93.4 ± 18.3	91.7 ± 12.4	141.8 ± 26.7	0.237	<0.001	0.224
MnSOD (%)	100.0 ± 19.0	109.2 ± 22.5	127.3 ± 16.7	147.1 ± 20.1	0.358	0.916	0.759

Abbreviations: CAT, catalase; SOD, superoxide dismutase; TLR4, Toll-Like Receptor 4; GPX: glutathione peroxidase; Cu,ZnSOD: copper zinc superoxide dismutase; MnSOD: manganese superoxide dismutase; Nrf2: nuclear factor erythroid 2-related factor 2. Results are expressed as mean ± SEM. Statistics by two-way ANOVA: AMD effect of adherence to the Mediterranean diet, T effect of time, AMD × T interaction between adherence to the Mediterranean diet and time. * Difference in means between participants under median value and above median value (AMD). # Difference in means between time. Each variable was adjusted by baseline values of the analyzed variable.

**Table 6:** Glutathione determination in blood of participants “Baseline” and “6 months” stratified by adherence to Mediterranean diet (AMD).

	Under Median Value (<50%)		Above Median Value (≥50%)		ANOVA		
	Baseline (n = 31)	6 Months (n = 31)	Baseline (n = 36)	6 Months (n = 36)	AMD	T	AMD × T
	Mean ± SEM	Mean ± SEM	Mean ± SEM	Mean ± SEM			
tGSH (mM/L blood)	3.27 ± 0.35	4.33 ± 0.28	3.27 ± 0.21	4.99 ± 0.25 #	0.237	**<0.001**	0.224
GSSG (mM/L blood)	0.345 ± 0.011	0.370 ± 0.011	0.342 ± 0.012	0.386 ± 0.014	0.585	**0.007**	0.457
tGSH/GSSG	9.81 ± 1.24	10.1 ± 0.78	11.9 ± 0.83	13.7 ± 0.96 #	0.279	**0.003**	0.409

Abbreviations: tGSH, total glutathione; GSSG, oxidized glutathione; tGSH/GSSG, ratio of total glutathione and oxidized glutathione. Results are expressed as mean ± SEM. Statistics by two-way ANOVA: AMD effect of adherence to the Mediterranean diet, T effect of time, AMD × T interaction between adherence to the Mediterranean diet and time. # Difference in means between time. Each variable was adjusted by baseline values of the analyzed variable.

**Table 7:** Immunoassays in plasma of participants “Baseline” and “6-month intervention” stratified by adherence to Mediterranean diet (AMD).

	Under Median Value (<50%)		Above Median Value (≥50%)		ANOVA		
	Baseline (n = 31)	6 Months (n = 31)	Baseline (n = 36)	6 Months (n = 36)	AMD	T	AMD × T
	Mean ± SEM	Mean ± SEM	Mean ± SEM	Mean ± SEM			
Immunoassays							
MPO (ng/mL)	3.87 ± 0.32	3.22 ± 0.20	4.57 ± 2.58	3.26 ± 0.15 #	0.153	<0.001	0.201
Resolvin D1 (pg/mL)	133.7 ± 4.5	156.6 ± 6.1 #	145.6 ± 3.82	162.7 ± 4.01 #	0.055	0.000	0.527
Irisin (ng/mL)	108.6 ± 10.0	98.3 ± 10.3	127.4 ± 11.6	109.3 ± 9.2	0.156	0.177	0.708
CK-18 (U/L)	60.5 ± 5.3	47.7 ± 5.3	78.2 ± 6.0	48.2 ± 2.9 #	0.073	<0.001	0.089
IL-6 (pg/mL)	4.27 ± 0.05	4.18 ± 0.05	4.31 ± 0.03	4.20 ±0.3 #	0.410	0.013	0.723
TNFα (pg(mL)	4.10 ± 0.20	4.03 ± 0.18	4.02 ± 0.07	3.93 ± 0.07	0.484	0.548	0.938

Abbreviations: MPO, myeloperoxidase; CK-18, cytokeratin 18; XOD, xanthine oxidase; IL-6, interleukin-6; TFNα, tumor necrosis factor alpha. Results are expressed as mean ± SEM. Statistics by two-way ANOVA: AMD effect of adherence to the Mediterranean diet, T effect of time, AMD × T interaction between adherence to the Mediterranean diet and time. # Difference in means between time. Each variable was adjusted by baseline values of the analyzed variable.

## Data Availability

There are restrictions on the availability of data for this trial due to the signed consent agreements around data sharing, which only allow access to external researchers for studies following the project purposes. Requestors wishing to access the trial data used in this study can make a request to pep.tur@uib.es.
